# On the Variability of the Dmanisi Mandibles

**DOI:** 10.1371/journal.pone.0088212

**Published:** 2014-02-20

**Authors:** José María Bermúdez de Castro, María Martinón-Torres, Mark Jan Sier, Laura Martín-Francés

**Affiliations:** 1 Dental Anthropology Group, National Research Center on Human Evolution (CENIEH), Burgos, Spain; 2 Paleomagnetic Laboratory ‘Fort Hoofddijk’, Department of Earth Sciences, Faculty of Geosciences, Utrecht University, Utrecht, The Netherlands; 3 Human Origins Group, Faculty of Archaeology, Leiden University, Leiden, The Netherlands; University of Kansas, United States of America

## Abstract

The description of a new skull (D4500) from the Dmanisi site (Republic of Georgia) has reopened the debate about the morphological variability within the genus *Homo.* The new skull fits with a mandible (D2600) often referred as ‘big’ or ‘enigmatic’ because of its differences with the other Dmanisi mandibles (D211 and D2735). In this report we present a comparative study of the variability of the Dmanisi mandibles under a different perspective, as we focus in morphological aspects related to growth and development. We have followed the notion of modularity and phenotypic integration in order to understand the architectural differences observed within the sample. Our study reveals remarkable shape differences between D2600 and the other two mandibles, that are established early in the ontogeny (during childhood or even before) and that do not depend on size or sexual dimorphism. In addition, D2600 exhibits a mosaic of primitive and derived features regarding the *Homo* clade, which is absent in D211 and D2735. This mosaic expression is related to the location of the features and can be explained under the concept of modularity. Our study would support the possibility of two different paleodemes represented at the Dmanisi site. This hypothesis has been previously rejected on the basis that all the individuals were constrained in the same stratigraphic and taphonomic settings. However, our revision of the complex Dmanisi stratigraphy suggests that the accumulation could cover an undetermined period of time. Even if “short” in geological terms, the hominin accumulation was not necessarily synchronic. In the same line we discard that the differences between D2600 and the small mandibles are consequence of wear-related dentoalveolar remodeling. In addition, dental wear pattern of D2600 could suggest an adaptation to a different ecological niche than the other Dmanisi individuals.

## Introduction

**Figure 1:**
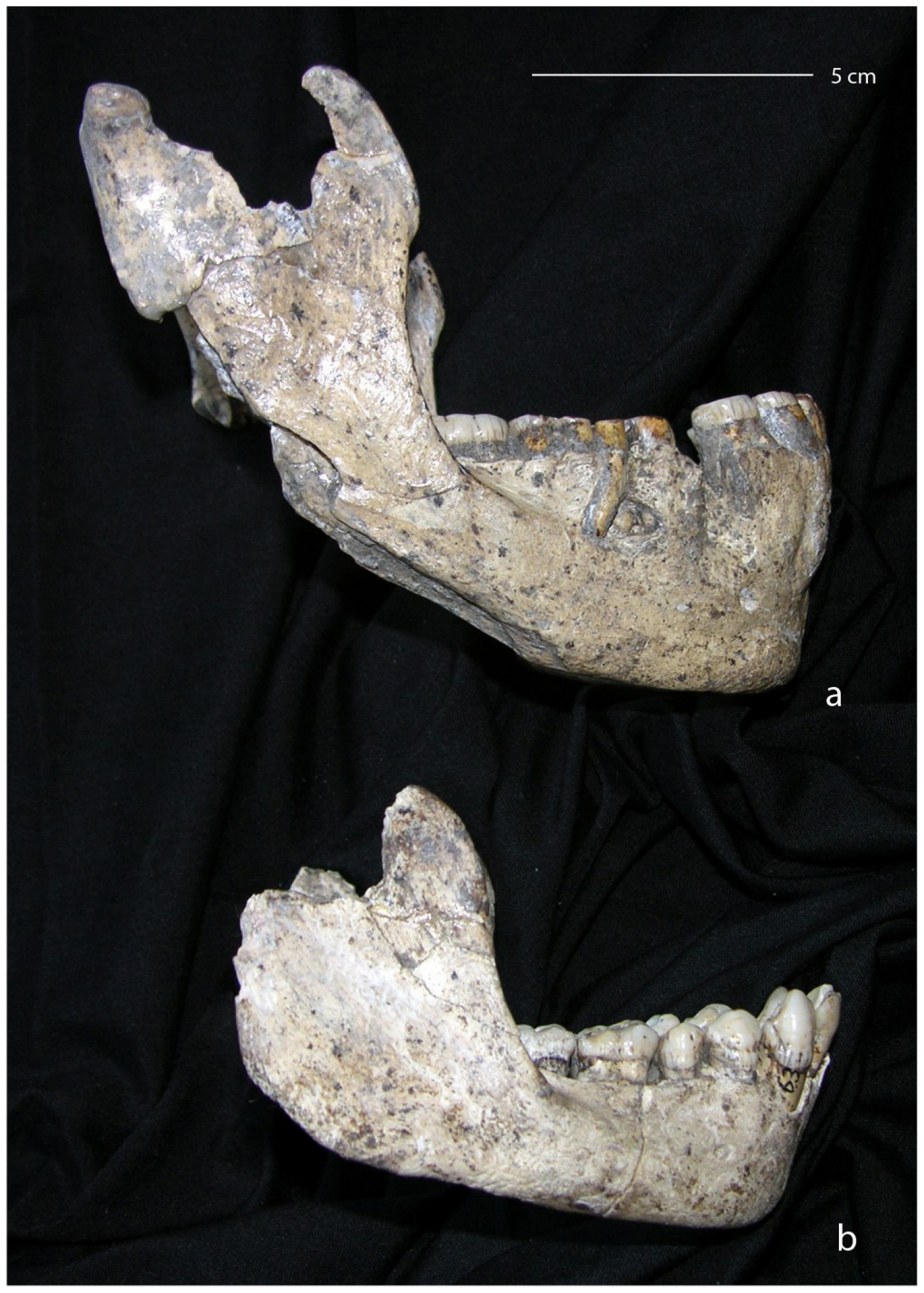
Lateral view of two Dmanisi mandibles. On top the D 2600 mandible (a), also referred as the “big mandible”, and below the D2735 mandible (b). Photo courtesy of D. Lordkipanidze.

The Pleistocene site of Dmanisi, in the south-eastern region of the Republic of Georgia, has yielded an impressive hominin fossil assemblage that dates back to 1.81–1.77 million years ago (Ma) [1]–[3]. The new evidence from Dmanisi has opened new perspectives for understanding the nature of the first Eurasian human settlers, and it has provided an important amount of data for a reassessment of the origin and evolution of the genus *Homo* [1], [4]–[11]. The Dmanisi hominins have been attributed to *H. erectus* [1], [11], [12], late *H. erectus* [13], *H. sp. indet (aff. ergaster*) [14], *H. ex gr. ergaster* [4], *H. georgicus* [15] and *H. erectus ergaster georgicus* [11]. The attribution to *H. georgicus* was based on the size and shape of one of the mandibles, D2600, found in 2000 (Figure 1a). This specimen is considerably larger than the other mandibles recovered from the site (D211 and D2735), and fits with a newly described skull (D4500) [11]. Under the light of the new fossil, also called skull 5, Lordkipanidze et al. [11] consider that the variability of the Dmanisi hypodigm can be accommodated in a single evolving lineage, which they identify with the taxon *H. erectus*. In this context, the Dmanisi population is formally designated as *H. erectus ergaster georgicus*.

Most researchers have acknowledged the high degree of morphological variability within the Dmanisi hypodigm, although their interpretations differ. Gabunia et al. [15] suggested that the variability could be explained by a marked sexual dimorphism. However, using the height and breadth of the mandibular corpus, Skinner et al. [16] compared the size and shape of D2600 and D211 and concluded that the pattern of variation did not resemble that of any living hominoid and was significantly larger than that of modern human populations. For Skinner et al. [16] the presence of a second hominin species in Dmanisi, represented by D2600, should be considered. The dental morphometric study also opens the possibility to the presence of two different populations at the Dmanisi site [10]. Rightmire and colleagues [12] also made comments about the “large and somewhat enigmatic individual documented by the D2600 mandible”, but rejected the hypothesis of two populations claiming that preservation of the mandibles was not good enough for this assessment [17]. However, they also recognized that if they belong to the same taxon, Dmanisi hominins would have a sexual dimorphism greater than that observed in modern humans and chimpanzees, and that the degree of intrapopulational variability would be similar to that of gorillas [17]. Also, according to these authors, it could be that the Dmanisi populations represent a novel dental and morphogenetic variation within the genus *Homo*. There are also claims [17], [18] that pathologies might have obscured features that could be taxonomically informative and that D2600 would have increased its size due to continuous growth because of age and as a response to a severe dental wear. The last study on the Dmanisi variability has been published by Lorkipanidze et al. [11]. These authors conclude that craniomandibular shape variation in the Dmanisi hypodigm is congruent with patterns and ranges of variation found in two chimpanzees species (*Pan troglodytes* and *P. paniscus*), as well as in a contemporary *H. sapiens*. According to Lordkipanidze and his team, the large variability exhibited by the Dmanisi hominins would lessen the differences that have been so far used to identify species such as *H. habilis*, *H. rudolfensis*, *H. ergaster* or *H. erectus* in the fossil record. All of these would thus belong to the same species, representing regional variants of a single *Homo* lineage that would have inhabited the Eurasian and African continents during a considerable large period [11].

Since the metric comparative study of the Dmanisi mandibles has been published before [16], [17], [19], this contribution is aimed to revise the morphology of the Dmanisi mandibular sample (excluding the toothless mandible D3900 which is significantly remodeled) from a different perspective. Beyond classic morphometric studies, we approach the Dmanisi mandibles considering that their architecture is the result of the developmental processes that occurred during the growth of the mandible. The understanding of these processes could help to explain the presence of some ‘enigmatic’ mandibular features and the variation observed in the Dmanisi sample. This study also wishes to contribute to the debate about the possibility of more than one hominin lineage represented in the Dmanisi hypodigm, a discussion that cannot be considered solved only because all the fossils have been found allegedly in the same level. The Dmanisi sample is too important to understand the origin and first stages of the genus *Homo* and the debate must continue.

### Previous conceptual considerations

#### Modularity and phenotypic integration

There are already several morphometric comparisons of the Dmanisi mandibles [12], [13], [15], [17]. Although we also compare some metrical and morphological features, our analysis is focused on a different perspective, taking into consideration aspects related to growth and development. Thus, the present analysis has been conceived under the notion of modularity, that is, “the division of biological structures, development, and physiology into standardized and repeatable units”, p. 116 of [20]. Every module could make none, one, or multiple functional roles [20]. The concept of modularity is essential in studies about complexity and phenotypic integration [21], [22], and can give a sound explanation to mosaic evolutionary patterns [23]. For instance, some studies support the notion that the corpus and ramus represent different developmental modules [24], [25].

We have some recent examples of the success on the application of these concepts to hominin evolution. González José and colleagues [26] made a study of the craniofacial complex in hominins under the assumption that functional and developmental integration leads to co-inheritance of the character complexes (modules), which are thus constrained to evolve in a coordinated fashion. This approach circumvents the difficulties arisen when we use discrete features in cladistic analyses at a lower taxonomical level [26]. These authors have considered only four craniofacial modules; each one including some presumably correlated variables. Their cladistic analysis of a large sample of hominins found that the subadult cranium D2700 from Dmanisi was associated with the clade formed by *H. erectus* and *H. ergaster*.

Polanski [27] concludes that the human mandible follows the same mammalian pattern of mandibular integration than other primates. This author has analyzed the morphological integration of the mandible in a longitudinal study of modern humans from the age of four to adulthood. Using some variables taken on radiographs and representing different regions of the mandible, Polanski [27] has found that the ascending ramus and the nonalveolar portion of the corpus remain integrated throughout ontogeny, with the weakest integration corresponding to the anterior corpus height. In contrast, the alveolar region of the corpus is dynamic, becoming modularized (non-integrated) during ontogeny, thus reflecting the need for space for the developing dentition. Some level of integration of this part of the mandible with other regions occurs during the adulthood. Other studies, which will be considered in other sections, have also used the concept of modularity to assess mandibular morphological variation [28], [29]. Of course, mandibular growth coordination (integration) between the different parts is essential for a correct biomechanical function. Furthermore, as it has been proposed by van Limborgh [30], genetic factors may also play an important role in growth and development, and it is well-known that there are a number of genetically encoded regulatory factors (like growth factors and homeobox genes), which can affect the morphogenesis of the craniofacial complex [31], [32].

#### The mandible as a set of integrated units

Although the mandible could be considered as an entity as such, it has been demonstrated that there is not necessarily a relationship among the size, shape, or position of the different parts of this skeletal unit, namely alveolar, basal, coronoid, angular, and condylar units [33], between corpus and ramus [34], [35], and even between the anterior and posterior portions of the dental arch [36]. The latter is in accordance with previous observations about the independent evolution of anterior and posterior dentitions [37], [38]. The reason for the relative independence between these parts of the mandible would rely on the fact that they accomplish different functions and thus, they can develop and evolve independently [36]. The growth of the alveolar component of the corpus appears to be clearly influenced by spatial requirements for housing the developing dentition, as well as for a correct occlusion with the maxillary dentition. Furthermore, the growth of the corpus is directly related to the growth of the nasomaxillary complex [39], [40], whereas the rami have a direct interplay with the pharyngeal space and middle cranial fossa [40], [41]. The shape of the coronoid process, which is very variable in the hominin fossil record, is influenced by the strength of the temporalis muscle, whereas the growth of the condyle and associated regions are strongly conditioned by their role in the articulation of the mandible with the temporal bone. In sum, the mandible can be considered as a set of relatively independent morphological units, whose development and evolution adapt to a particular architecture [42]. Furthermore, the morphology of the mandible, as the skeletal unity of a functional matrix, depends on some factors related to the craniofacial development [43]. The architectural structure of the mandible is the result of the interaction between its different components and between them and the craniofacial complex. Bone remodeling by resorption and deposition allows the relocation of a particular skeletal unity (such as the mandible and its different components) into its functional matrix [33].

The mandible includes two basic functional components, the corpus and the ramus. As we stated above, the mandibular corpus is influenced by spatial requirements for housing the developing dentition, and it is also a direct counterpart of the maxillary corpus in order to achieve a correct occlusion with the maxillary dentition. The ramus is related to the pharyngeal space and the middle cranial fossa, and his main function is to connect the corpus with the cranium by means of its articulation with the temporal bone. Moreover, there are several micro skeletal units form the mandible such as alveolar, coronoid, ramus or symphysis among others, and each of them is subjected to different genetic and environmental influences. The growth of the mandible and the alignment of its different components can be understood in the field of the growth of the soft tissues [44], [45].

In modern humans it has been demonstrated that the mandible grows in a downward and forward direction via posterior growth and anterior displacement [46]. Furthermore, the mandible suffers anterior and posterior rotations over the course of growth [47]. The anatomical features we observe in the dry bone are indeed the reflection of all these processes. Specific morphological features reflect a specific pattern of growth and development [48]. Obviously, the mandible of australopitecines, for instance, is different to that of modern humans, since we have a different morphogenetic growth. A review of the genetic factors involved in the mandibular growth and morphogenesis can be found in [49]. Summarizing, the spatial arrangement of the different mandibular parts is the result of a strong genetic and functional influence and reflect specific developmental patterns. Thus, we will pay a particular attention to the architectural relationship between the corpus and the ramus in our mandibular sample.

## Materials and Methods

### The dmanisi specimens

In this study we include the first mandible recovered the Dmanisi site, D211, the mandible D2735 (Figure 1b) that fits with the cranium D 2700 and the mandible D2600 (Figure 1a), which fits with the cranium D4500. Although D2735 belongs to a subadult, the upper M3 of D2700 completed gingival eruption and was close to occlusion. We are aware that a possible criticism to this study might the inclusion of D2735 as it belongs to a subadult individual and some parts may have not reached its adult shape. However, as we will explain below the variables we are focusing in are not affected by its age. It has been shown that early in ontogeny, when dental development is still in progress, the differences in mandibular shape at the genus level [50] and at the species level [51] are already established. Indeed, in these species, mandibular shape differences are reached near or before birth. More importantly for our study is that, according to Daegling [52], “… the spatial relationships between component parts (e.g. ramus and corpus) are established early in ontogeny and remain invariant during growth”. In modern humans, the definitive breadth of the mandible is reached early in ontogeny, since the maximum breadth of the cranium occurs at an age of about 2–3 years and no significant shape differences in the architecture of the mandible are expected after this age [40], [46], [53]. Thus, patterns in facial growth in modern human are established in early development [54]–[56]. Bastir and colleagues [57] showed the differences between Neandertals and modern humans during their postnatal ontogeny. The growth trajectory in these species produces drastic changes between the corpus and the ramus due to the vertical growth occurring at the anterior and posterior face [29], [58]. Bastir et al. [57] suggest that the marked differences in the mandibular ontogeny of Neanderthals and humans are probably related to differences in the anterior face. Furthermore, some studies have shown that the rate of remodeling and rotation of the mandible during the primary dentition and mixed dentition stages is greater than during adolescence, and might play an important role in the determination of adult skeletal and dental relationships [32], [59]–[61]. In sum, it is important to remark that despite that the growth of the different parts of the mandible is decoupled in a significant degree, the spatial relationship between these parts (i.e. the structural architecture), such as the corpus and ramus, are established early in ontogeny and remain invariant throughout later stages of development. Since the individual to whom D2735 is not far from reaching the adult stage we cannot expect significant changes in the architecture of this mandible that, indeed, is very similar to that of D211.

Finally, we approach the study of D2600 under the premise that its taxonomic assessment is not influenced by pathological processes [62]. According to Margvelashvili et al. [18], the corpus and the symphysis of D2600 would have increased their height to compensate the extreme dental wear. However, in general, compensatory mechanisms related with occlusal adaptation could provoke the recession and thickening of the alveolar crest but no its increase [63]–[65].

The data and observations we refer in this study were obtained in the original fossils held at the Georgian National Museum (Tbilisi). The permit to study the material was given by Dr. Lordkipanidze under the frame of the Cooperation Treaty between Fundación Duques de Soria and Georgian National Museum, Proyecto 10-CAP-0078 supported by Ministerio de Asuntos Exteriores (Agencia Española de Cooperación Internacional y Desarrollo-AECID) and Ministerio de Cultura of Spain. As we stated above, the edentulous D3900 mandible, which fits with the toothless D3444 cranium, was not included in this study, because the strong bone resorption has erased most of the morphological features of interest. In order to compare our observations, other hominin mandibles (originals and high quality first generation casts) have been used (see Table 1). For comparative purposes we have used data presented in Tables 3 and 4 of Rosas and Bermúdez de Castro [14] and Table 1 of Rosas and Bermúdez de Castro [66].

**Table 1:** Comparative sample included in this study (originals and high quality casts)*.

**Early Pliocene (Africa)**	AL188-2, AL266, AL277-1, AL288, AL400, Makapansgat MLD 18, Makapansgat MLD 40, Sterkfontein Sts 52
**Early Pleistocene (Africa)**	OH 13, KNM-ER 721, KNM-ER 730, KNM-ER 992, KNM-ER 1501, KNM-ER 1802, KNM-ER 1805, KNM-ER 3230, KNM-WT 15000, Swartkrans SK 15, Peninj
**Early Pleistocene (West Asia)**	Dmanisi D211, Dmanisi D2375, Dmanisi D2600
**Early Pleistocene (Europe)**	Gran Dolina TD6, ATD6-96
**Middle Pleistocene (Africa)**	KNM-BK 8518, KNM-BK 67, OH22, Tighenif 1, Tighenif 2, Tighenif 3
**Middle Pleistocene (East Asia)**	Zhoukoudian G1, Zhoukoudian H1
**Middle Pleistocene (Europe)**	Arago 2, Arago 13, Montmaurin, Atapuerca-SH: AT-1, AT-250, AT-300, AT-505, AT-605, AT-607, AT-888, AT-950, Mauer
**Early Upper Pleistocene (Europe)**	Amud 1 & 2, Vindija 226, Krapina J & H, Ochoz, Tabun 1 & 2, Spy 1, Regourdou, Circeo 3, Kebara, St. Cesaire, Shanidar 4, La Quina 1
**Upper Pleistocene (Europe)**	Abri Pataud,Combe Capelle, Skhul 5, Qafzeh 9, Pavlov, Predmostì, Kostienski, Ober 1 & 2, Brno 3, Dolni 3, Cromagnon, Chancelade

No taxonomical attributions are assumed in this table.

### Stratigraphic context of the dmanisi mandibles

Lordkipanidze et al. [11] state that all human fossils come from a spatially and temporally constrained stratigraphic and taphonomic setting, being this the premise to assume that all the Dmanisi individuals belong to the same paleodeme. Dmanisi hominins have been mostly found in two different excavations areas namely Block 1 (where D211, D2280, D2282 have been found) and Block 2 (where D2735, D2700, D4500 and D26000 have been found) separated about 10 to 20 m. Stratigraphy of Dmanisi is complex, including several units and “pipes” [6], [67] that were not recognized in the first stratigraphic studies. As a consequence, the interpretation and nomenclature of the Dmanisi stratigraphy has been changing throughout the last decades, making difficult to trace the specific origin and dating of each of the specimens as well as correlations among all the related publications.

**Figure 2:**
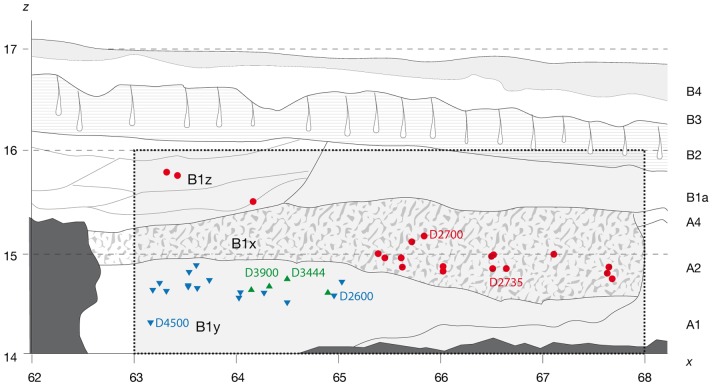
Projected stratigraphic location of the main hominin remains recovered from Block 2. Adult individuals composed by D2600 and D4500 (red) and D3900 and D3444 (green) are found in layer B1y. The adolescent individual composed by D2700 and D2735 (red) is recovered from B1x. A third adult individual is found at level B1z (red). Figure combined and modified from Figure 1b of Lordkipanidze et al. [9] and Figure S1–lower- from Lordkipanidze et al. [11].

Dmanisi stratigraphy was first divided into 7 units labelled with Roman numbers, with number I being the top unit, and number VII being the lowest, just above the Masavera basalt [68]. The second stratigraphic division of the same profile proposed 8 units labelled from B2b at the top (reverse polarity), to unit A1 (normal polarity) at the bottom [4]. Ferring et al. [69] expanded this division with units that are not present at the main profile. As stated above, Dmanisi stratigraphy is additionally complicated by the development of pipes, which are erosional or soilpiping features incised in unit A and filled up by material that, according to Rightmire et al. [12] is penecontemporary with B units. The sedimentary units within the pipes are labelled as B1y, B1x and B1z (but are not part of unit B1) from bottom to top and reflect distinct layers of sediment accumulation inside the pipes and gullies as it can be seen in Figure 2 of Lordkipanidze and colleagues [6]. In earlier works [1], [4], [70], several fossils (D2280, D2282, D211, D2700 and D2735) were attributed to unit V (or unit A), with normal polarity (i.e., Olduvai subchron). However, later on, Ferring et al. [69] stated, contra [1], [3], [4], [12], [70], [71], that “in the main excavations, no artifacts or fossils have been found in the older stratum A deposits, which conformably overlie the Masavera Basalt”. According to Ferring et al. [69] all human fossils have been recovered from reversely polarized strata B1x-B1z pipe and gully sediments. Even if we assume the latter as the correct stratigraphic interpretation, the attribution of all the hominin fossils to the same sedimentary layer is not supported. D2600, which was firstly attributed to unit VI (or unit A) by Gabounia et al. [19] and Lumley et al. [3] is now assigned to level B1y [72], together with its newly described skull D4500 [11] (see Figure 2). Also, the edentulous individual (D3900/D3444), firstly attributed to stratum B1 [5] is now located in unit B1y [6]. However, the adolescent individual composed by D2700 and D2735, firstly attributed to unit V (or unit A) by Vekua et al. [70] is assigned to B1x and thus, by definition, to a different sedimentary episode than D2600, D4500, D3900 and D3444 (Figure 2). Finally, an adult individual represented by a single metatarsal was found at the higher stratigraphical position (layer B1z) [67]. As stated by Lordkipanidze et al. [72] there is virtually no stratigraphical overlap between the postcranial remains of the adolescent individual (layer B1x) and those attributed to D4500/D2600 and D3444/D3900 (layer B1y) supporting the distinction and different accumulation moments. Thus, even if the cycles of gully construction and filling are allegedly “short”, less than 10.000 years according to Lordkipanidze and colleagues [72], human remains have been found in three different stratigraphic layers of pipe infilling covering an uncertain time of period.

Finally, we could not find the specific attribution of the human fossils found at Block 1 (D2280, D2282, and D211) to either B1x, B1y or B1z, so the precise stratigraphic and taphonomic setting of the complete set of human fossils remains to some extent unclear. In addition, it is accepted that the human accumulation is younger than the Olduvai subchron [69], [73] and according to some researchers it could be as young as 1.07 Ma [71].

Summarizing, even if we assume that all fossils come from the pipes [69], the different sublayers (y, x and z) suggest that the hominins could be deposited there directly, could be re-deposited from sediments of different age or could be a combination of both processes, meaning that they are most likely not synchronic.

### Morphological features

Previous works provide a complete list of morphological features in the Dmanisi mandibles [1], [7], [12], [13], [19], [70], [74]. Our study focuses on some morphological features (Table 2), which can shed light on the growth model of the Dmanisi mandibles and help to test the null hypothesis that all the specimens belong to the same paleodeme.

**Table 2:** List of mandibular morphological features considered in this study.

Mandibular features
Shape of the alveolar arcade.
Presence/absence of mental protuberance, mental fossae, and lateral tubercles.
Presence and place of the anterior marginal tubercle.
Superior transverse torus.
Alveolar prominence.
Inferior transverse torus.
Place of mental foramen.
Place and size of lateral prominence.
Presence and trajectory of the mylohyoid line in relation to alveolar margin.
Relief of masseteric fossa.
Presence/absence of the retromolar area. Position of M3 in relation to ramus.
If present, inclination of the retromolar area.
Shift between the anterior and posterior parts of the dental arcade.
Spatial relationship between the corpus and ramus.
Breadth of the sulcus extramolaris.
Shape of the subalveolar plane and subalveolar fossa.

### Supporting metrical variables

Not all the measurements could be taken in all specimens since D211 lacks the two rami and D2735 is an immature individual. However, the following metrical variables and indexes can help to support the morphological observation.

Breadth of the corpus at the level of the lateral prominence.
Breadth of the sulcus extramolaris.
The distance between the more prominent points of both the right and -left lateral prominences (LP-LP).
Width: bi-molar M3-M3 distance [14].
Length: infradentale (ID)-M3 distance [14].
Total length of the alveolar arcade: LAA. L7 in [19].
Condylion-Menton distance (CON-MEN). The condylion landmark is defined as the uppermost point of the condyle, whereas the menton is the lowermost point of the mandibular symphysis on the mid-sagittal plane [66].
Minimum breadth of the ramus.
Distance between the mental foramen and the third molar (FOR-M3) [66].
Distance between the foramen mental and the infradental point (FOR-I) [66].
Distance between the M3 and the lingula (LIN) [66].
Angle between the line defining the internal alveolar border of the corpus (level of M2–M3) and its prolongation in the internal border of the ramus, which coincides with the crista endocoronoidea. This angle has been obtained from occlusal photographs of the specimens so that the two lines defining it are placed in the same plane.

In relation to the latter, it is interesting the particular comparison with the sample recovered from the Middle Pleistocene Atapuerca-Sima de los Huesos site. This sample has been recovered from a same level and very probably belongs to the same biological population. It is, therefore, the best hominin sample to compare with the Dmanisi hypodigm and explore the degree of intrapopulational variability.

## Results

### Common Features of the Dmanisi Mandibles

**Figure 3:**
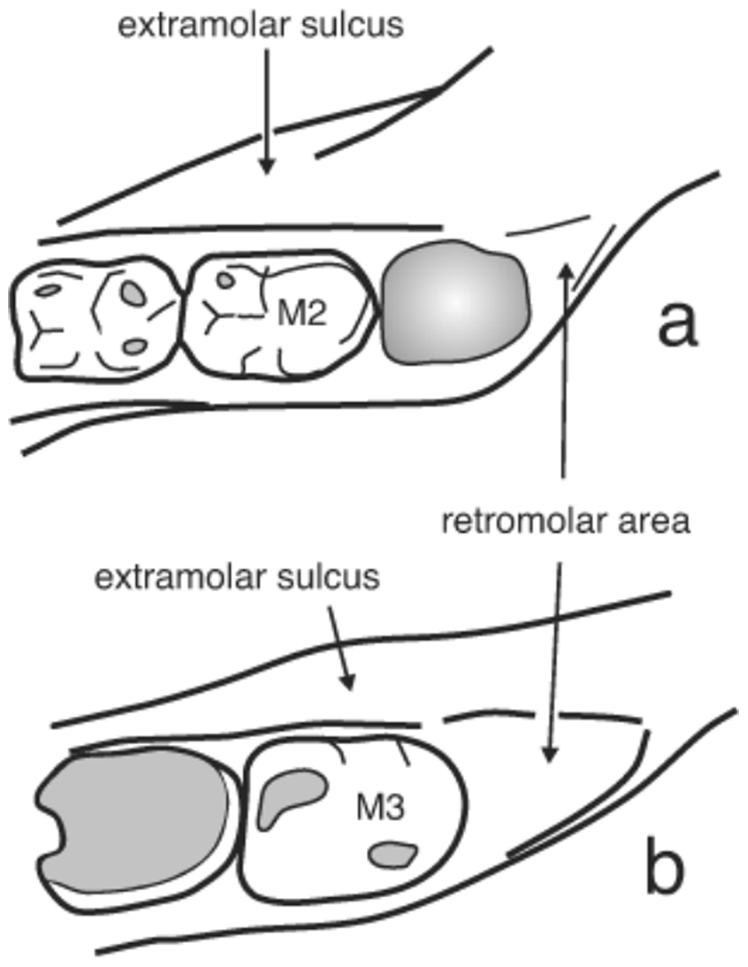
Anterior view of D2600. Note the forward position of the anterior marginal tubercles at the level of the canines, the large canine eminences, and the clear mental protuberance.

The three Dmanisi mandibles present a set of common primitive traits for the *Homo* clade, namely:

Position of the main mental foramen at the level of P3–P4. In D211 the mental foramen is slightly advanced with respect to the other specimens, at the level of the distal part of P3.
Anterior marginal tubercle placed at the level of the canine (D 2600), or at the level of C-P3 (D211, and D2735). This feature seems to have appeared for the first time in the *Homo* clade [14], [75], [76]. According to Rosas and Bermúdez de Castro [14], the anterior marginal tubercles began their evolutionary differentiation below the canine in early *Homo* and then migrated to more distal positions. The Dmanisi mandibles seem to confirm this hypothesis.
Presence of features on the anterior part of the symphysis (Figure 3). D211 exhibits a noticeable and prominent mental trigone, but without signs of mental fossae or lateral tubercles [14]. In D2735 there is a clear mental protuberance between the alveolar border and the base, although it is less defined than in D211. In D2600 the external aspect of the symphysis is vertical. Its morphology is in part obscured by a pathological process (abscess), including the presence of a loss of bone at the base of the right lateral incisor with clear signs of remodeling [62]. The canine eminences are large and very conspicuous, also giving a peculiar aspect to the external face of the symphysis. However, we can also observe a faint mental protuberance in this specimen (but see below). The presence of a well-developed mental trigone is considered as a derived feature in the *Homo* clade [13], [14], [77], [78].
D211 and D2600 share a high value for the alveolar arcade index (ID-M3/M3–M3 distance ×100). In D211 the index is 110.2 (66.7/60.5) and in D2600 the index is 108.2 (76.4/70.6). The high index in these specimens reflects the U-shaped dental arcade of these mandibles, a primitive trait shared with many early African hominins, like AL 288, AL 400, OH 13, KNM-ER 1805, KNM-ER 992, KNM-BK 8518, and KNM-BK 67 [14]. The absence of third molar in D2735 prevents the possibility of obtaining the index in this specimen. However, the U-shaped dental arcade of D2735 is evident at a first sight.
In consonance with previous features, the shift of direction between the anterior and posterior parts of the dental arcade occurs at the level of the canine in the three Dmanisi specimens.
The superior transverse torus is rounded and prominent. It crosses the internal aspect of the symphysis from one side to the other and extends laterally into a conspicuous alveolar prominence, which gradually decreases in thickness and ends at the level of M2. It forms a remarkable bulge in D2600 and D2735 at the level of P4. The formation of a laterally expanded lingual arcade in the Dmanisi specimens represents a reminiscence preserved in early *Homo* mandibles, like OH 13, OH 37, KNM-ER 992, and KNM-ER 15000.
The torus mandibularis is well-developed in the three Dmanisi mandibles, but it is especially marked in the small specimens, D211 and D2735.

### Differential Features in the Dmanisi Mandibles

**Figure 4:**
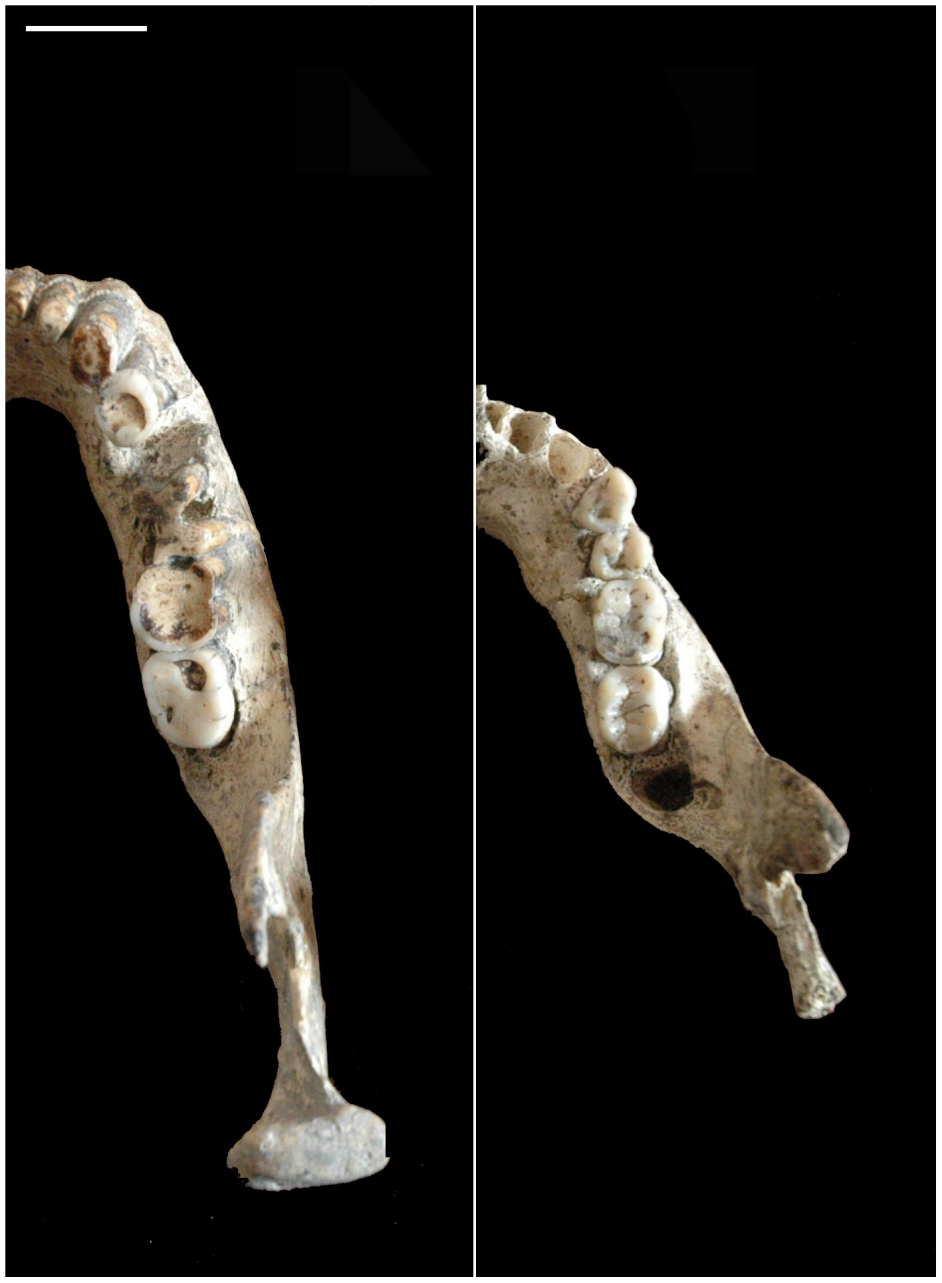
Scheme of the distal part of the *corpus* of D 2735 and D 2600. Note how the internal alveolar border, at the level of the mandibular tuberosity, turns buccalwards just behind the M3, as well as the small size of the retromolar area in D2735 (a) in relation to D2600 (b). The different spatial relationship between the corpus and ramus in the two mandibles has an influence on the breadth of the corpus at both the lateral protuberance level and the extramolar sulcus.

**Figure 5:**
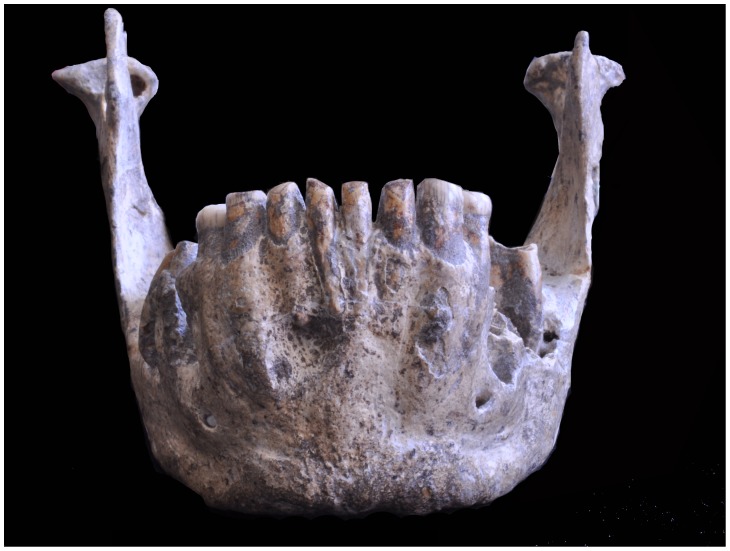
Detail of the spatial relationship between the corpus and ramus in D2600 and D2735. Note that the ramus is wholly aligned with the corpus in D2600 (left) and displaced backwards, whereas in D2735 (right) the ramus deviates strongly buccalwards (see the text for more details). White bar: 2 cm.

The small specimens, D211 and D2735, share some features that differ from those of D2600, namely:

In spite of the resemblances in the external part of the symphysis (see above), it is interesting to note the presence of lateral tubercles near the mental protuberance in D2600 and its exaggerated height of the corpus at this level (49,0 mm), in contrast to 30,8 mm in D211 [16] and 32,3 mm in D2735.
In D211 and D2735, the inferior transverse torus is less projected than in D2600 and it is hidden by the superior transverse torus when we observe the mandible from the upper view, like in all other early *Homo* specimens. Gabunia et al. [4] note the remarkable distal projection of the inferior transverse torus in D2600, a primitive feature that this specimen shares with *Australopithecus* and *Paranthropus.*
The mylohyoid line is diffuse in D211 and absent in D2735, a primitive trait of the *Homo* clade, also shared with the *Australopithecus* genus [79]. In contrast, D2600 exhibits a conspicuous mylohyoid line, which begins at the end of the crista endocoronoidea and descends obliquely and ends up in the inferior transverse torus. The value of this index in several hominin specimens can be found in Table 3 of [19]. At the level of the M3, the distance between the mylohyoid line and the alveolar border is 7.8 mm. The diagonally oblique position of the mylohyoid line close to the alveolar margin at the M3 level is a typical Neandertal feature (see discussion), and it has been considered an apomorphy in these hominins [48]. This feature is also present in some of the mandibular specimens from the Sima de los Huesos site of Atapuerca, which are related to the Neandertal lineage. An inclined mylohyoid line is also present in SK45 and Thigenif 3. However, these examples (and with the exception of Neanderthals and related hominins) seem to be exceptional in the hominin fossil record [48].
The sulcus extramolaris is wide in the three mandibles, but it is relatively wider in D211 (9.6 mm) and D2735 (10.1 mm) despite they are considerably smaller than other variables. In D2600 the sulcus extramolaris is 8.5 mm wide.
The lateral prominence is well marked in D211 and very prominent in D2735 and placed at the level of M1–M2, whereas in D2600 it is weak and located at the level of M2–M3. The breadth of the corpus at this level is about 20.0 mm in D211, 23.5 mm in D2735. Although in D2600 the breadth of the corpus at the level of the lateral prominence is 22.7 mm, it is important to consider this value in relative terms by considering other variables of the specimens, such as the height of the corpus [16]. Thus, we have also obtained the breadth between the more distant points of the right and left LPs. The respective values of the latter are: 83.5 in D2600, 80.4 in D2735, and 75.4 in D211. This measurement can be assessed in relative terms using the total length of the alveolar arcade (LAA). We have used the values obtained by Gabounia et al. [19] for D2600 (73.0) and D211 (62.0), and we have estimated this value in D2735 (63.0). The LP-LP/LAA-LAA index is 78.3 in D2735, 82.2 in D211, and 87.4 in 2600. These values clearly indicate the relative great breadth of the small mandibles at the level of the lateral prominence in comparison to D2600.
The main difference between the small mandibles and D2600 is related to the spatial relationship between the *corpus* and the *ramus* (Figures 4 and 5) and both the lateral prominence and the sulcus extramolaris are features related to this. This spatial relationship was already analyzed by Rosas and Bermúdez de Castro [14] in their study of the D211 specimen. The primitive pattern is characterized by the following traits: the sagittal plane of each *ramus* is placed in a buccal position in relation to the sagittal plane of the corresponding (right or left) part of the *corpus*. Furthermore, the anterior border of the *ramus* grows anteriorly reaching a forward position and conforming a conspicuous lateral prominence, with its maximum breadth located at the level of M1–M2. As a consequence, a wide extramolar sulcus is developed and the M3 and even a part of the M2 are covered by the *ramus*, when the mandible is observed from a lateral view. We also measured the angle formed by the internal alveolar border of the corpus at the level of M2–M3 (see methods) and the prolongation of this border in the ramus along the crista endocoronoidea in a representative hominin sample. The two lines converge at the level of the lingual tuberosity, an important area for mandibular growth, and that ought to be in line with the maxillary tuberosity for a correct dental occlusion, forming the so-called maxillary plane or PM plane [32]. The value of this angle in D211 is 120°. In D2735 the angle measures 130°, whereas in D2600 the value of this variable is 155°. Observations in the hominin fossil record seem to confirm that the buccal position of the ramus with respect to the corpus is the primitive condition in the hominin clade, as it is evinced in *A. anamensis*, *A. afarensis*. *A. africanus*. *P. africanus*, *P. boisei*, and *H. habilis* [14], [80]–[84]. Table 3 presents the value of the angle between the internal alveolar border of the corpus and the prolongation of this border in the ramus. Note that the smaller measurements are obtained in early hominins, and that the value in D211 is close to the lower limit of the variation of this angle in the hominin sample we selected. In representatives of *H. ergaster* (or African *H. erectus*) the values are higher and similar to that obtained in D2600. It is also interesting the low variation observed in the Atapuerca-SH hominin sample (coefficient of variation = 2.8). This sample includes specimens of great size, which may be considered as males, and small specimens which may represent females. Thus, SH is a good sample to show that differences in this architectural pattern are not necessarily related to sexual dimorphism or size differences. The Atapuerca-SH hominins form part or are closely related to the Middle/Upper-Pleistocene hominin lineage, which exhibit a extremely derived state showing the rami wholly aligned with the corpora and displaced backwards [66]. D211 and D2735 exhibit the primitive state for the spatial relationship between the *corpus* and *ramus*, whereas in D2600 the rami are wholly aligned with the corpora and displaced backwards.
The morphology of the retromolar area is also closely related to the spatial relationship between the *corpus* and *ramus*. In the small mandibles the internal alveolar border twists sharply buccalwards just behind the M3 and, as a consequence, the retromolar area is absent (D211) or poorly developed (D2735). In D211, the triangular torus arises just behind the M3 [14], whereas in D 2735 there is a narrow and vertical retromolar space, which is 7.6 mm long. In D2600 the retromolar area is sub-horizontal and measures 10.0 mm.
Also related to the spatial relationship between the *corpus* and *ramus* is the aspect of the subalveolar plane and the subalveolar fossa (the medial wall of the *corpus*) at the level of M2 and M3. In both D211 and D2735 the plane is flat and the fossa inclined and strongly inclined outwards, respectively. This inclination is necessary to get the anatomical connexion between the two components of the mandible, and it produces a conspicuous lingual tilt of the M3. This feature is also present in the African specimens KNM-ER 992 and KNM-ER 15000, as well as in Sangiran 9. In contrast, the mandible D2600 shows a flat, wider and almost vertically oriented subalveolar plane and subalveolar fossa. The later seems to be a derived condition of the *Homo* clade, which is present, for instance, in the Tighenif mandibles [85]. Furthermore, the pterygoid fossa is well-excavated in D2600.
In D2735 the masseteric fossa is deep, probably due to the eccentric position of the *ramus* with respect to the sagittal central axis of the mandible. The D2735 specimen shares this primitive structural pattern with early hominins like AL 400, AL 228, MLD 18, MLD 40, or OH 13. Although both the left and right ascending rami of D2600 are seriously damaged, it is possible to assess the masseteric fossa in the right side, which is very shallow.
Mounier and colleagues [74] have noted additional differences between D211 and D2600 such as that the incisura submentalis is present in D211 and absent in D2600. The latter shows a medial crest between the two fossae digastrica, which is absent in D211. Likewise, D2600 exhibit a conspicuous sulcus intertoralis, which is absent in D211.

**Table 3:** Measurement of the angle formed between the internal alveolar border of the corpus and its prolongation in the internal border of the ramus.

Specimen/Sample	Degrees	
Dmanisi D211	120	
Dmanisi D2735	130	
Dmanisi D2600	155	
MLD 18	127	
MLD 40	115	
Sts 52	148	
SK 15	139	
Peninj	130	
KNM-ER 721	148	
KNM-ER 992	151	
KNM-WT 15000	130	
OH 13	128	
OH 22	152	
ATD6-96	150	
Tighenif 1	155	
Tighenif 2	143	
Tighenif 3	145	
Mauer	155	
Atapuerca-SH	159,5 (n = 8; SD = 4,47; range = 154–166 )	

This angle measures the spatial relationship between the corpus and ramus. The Atapuerca-SH sample includes the mandibles of individuals I, IV, VI, XII, XIX, XXI, XXII, and XXIII.

A summary of the state of expression of the main features observed in D211, D2735 and D2600 is presented in Table 4.

**Table 4:** Summary of the primitive mandibular features in the *Homo* clade considered in this report.

**Primitive features in the ** ***Homo*** ** clade**
1- Mental foramen placed at the level of P3–P4
2- Presence of mental protuberance
3- Anterior marginal tubercle at the level of the canine or the C/P3
4- U-shaped arcade: high value for the alveolar arcade index
5- Shift between the anterior and posterior parts of the dental arcade at the level of the canine
6- Conspicuous alveolar prominence
7- Prominent superior transverse torus
8- Absence of retromolar area
9- Non-aligned corpus and ramus
10- Absence of mylohyoid line
11-Lateral prominence placed at the level of M1–M2
12- Subalveolar fossa strongly inclined outwards
13- Deep masseteric fossa
14- Anterior order of the ramus covering the M3 and a part of the M2
**Derived features in D2600**
8- Presence of a subhorizotal and well-developed retromolar area
9- Aligned corpus and ramus
10- Conspicuous and inclined mylohyoid line
11- Lateral prominence placed at the level of M2–M3
13- Shallow masseteric fossa
14- Anterior border of the ramus displaced forward and covering only a part of the M3

Traits 1 to 7 are common to D211, D2735 and D2600. However, traits 8 to 14 are derived in D2600.

### Some mandibular measurements and indices

In D2600 the distance between the CON (condylion) and MEN (menton) is 159.0. This is the greatest value obtained in a large sample of hominin mandibles [66]. This measurement, as well as the distance between the mental foramen and the M3 (FOR-M3∶61.8) are out and far from the variability of hominin groups like *H. erectus*, *H. ergaster* or *H. habilis*. With regard to the FOR-M3 distance, only two specimens are close to the value obtained in D2600: AL266 (59.9), and AL277-1 (59.3). This result accounts for the great length of the posterior part of the corpus, which gives body to molars and premolars. The large retromolar area of D2600 is a plus for this length. In D211, the distance FOR-M3 (50.0) is similar to most African and Asian Plio-Pleistocene hominins [66].

Likewise, and although the breadth of the ramus of D2600 (44.4 mm) is well within the variability of *H. ergaster, H. erectus*, or *H. heidelbergensis*, when this measurement is compared with the length of the mandible, the index ramus breadth/CON-MEN (0.28) is out and far from the variability of all the hominins except *H. sapiens*, only entering in the lower range of modern humans variation [66]. The distance between the infradental point and the mental foramen (I-FOR) has been used by Rosas and Bermúdez de Castro [66] to evaluate the taxonomical status of the Mauer mandible. For this measurement (39.8), D2600 is far from *H. habilis*, *H. ergaster* and most *H. erectus* [66]. In D211 the I-FOR distance (29.0) is similar to that of African earlier specimens including in *A. afarensis*, *H. habilis* and *H. ergaster* as well as in the East African Middle Pleistocene specimens from Baringo (BK67 and BK8518) [66]. The value of D2600 is well within the Middle European hominins. Concerning the distance M3-LIN (32.0), D2600 is also far from *H. habilis*, *H. ergaster*, and *H. erectus*. Only the values of Tighenif 3, BK67, OH22 and the European Middle Pleistocene hominins have similar values to that of D2600. When we compare this variable with the total length (M3-LIN/CON-MEN) index (0.2) D2600 is also out of the *H. erectus* variability [66].

### Some dental traits

A detailed study of the morphological and metric features of the Dmanisi dental sample can be found in Martinón-Torres et al. [10]. However, it is interesting to remember some aspects of the Dmanisi teeth that are relevant for the conclusions of this study. Thus, it is noteworthy that D211 and D2735 represent the first occurrence of an M1>M2 size sequence in the genus *Homo* record, whereas D2600 keeps the primitive pattern (M1<M2<M3) (see Figure 5). The M1>M2 sequence is a trait is not found again till the Middle Pleistocene in specimens such as OH-22, Rabat, Atapuerca-SH or some Zhoukoudian individuals, although is still infrequent during this period. In addition, D211 exhibits an M2>M3 size relationship like in OH-16, KNM-ER 806, KNM-ER 730 y KNM-ER 992, and some other specimens assigned to *H. erectus* and *H. antecessor* [38]. In contrast, D2600 shares the primitive pattern with *Australopithecus, Paranthropus* and the majority of the early *Homo* specimens (M2<M3).

**Figure 6:**
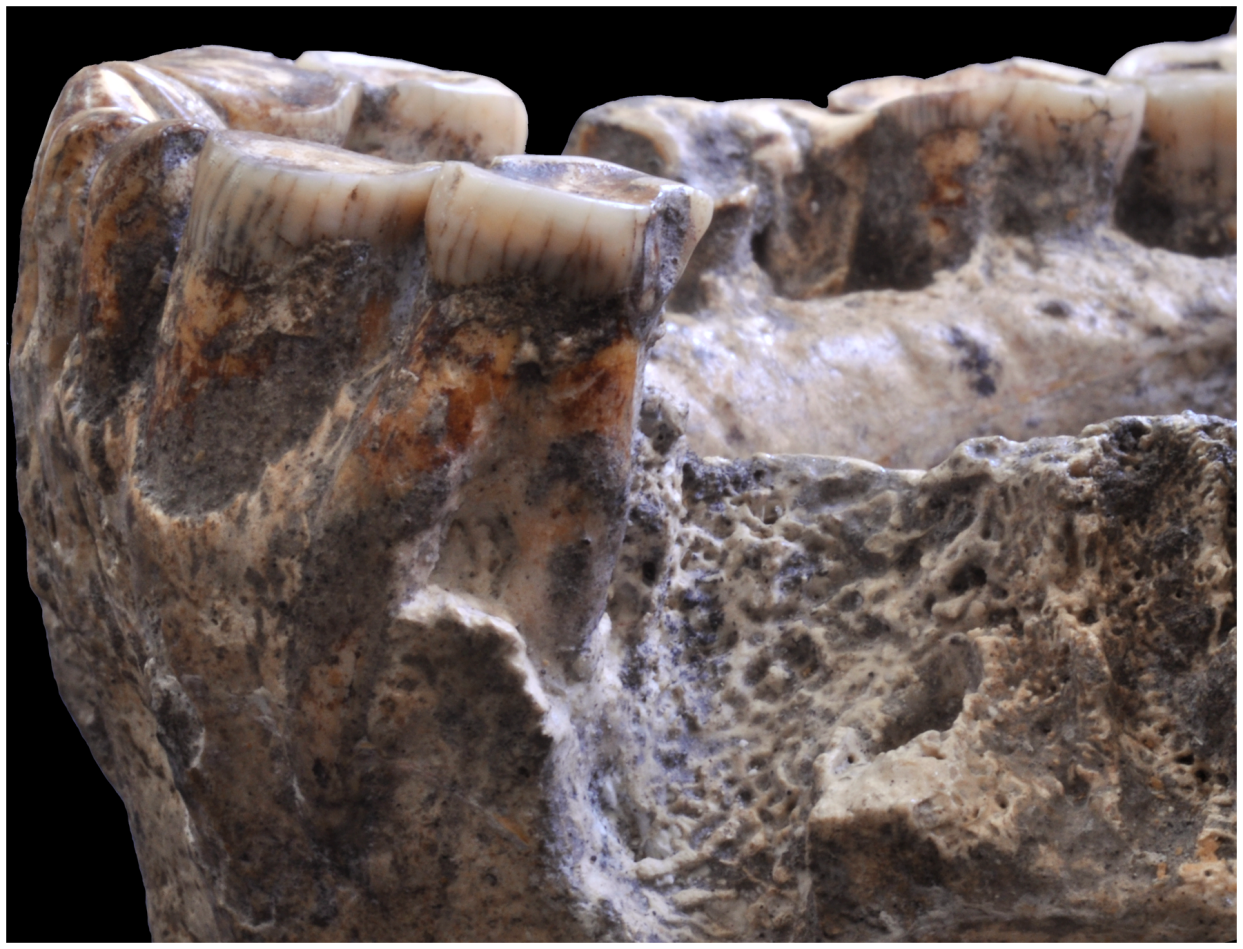
Detail of the left side of D2600. Note the molarized root bifurcation of the left P3.

The root system also illustrates differences between D211 and D2735, on the one hand, and D2600 on the other. D211 and D2735 exhibit a single root in their premolars, whereas D2600 develops a 2R: M+D pattern in its P3s (Figure 6) like in KNM-ER 1802 and UR-501 as well as in *P. robustus and P. boisei* [86].

## Discussion

In general, all researchers acknowledge large differences, not only in size but also in shape, between D2600 and the other two Dmanisi mandibles (D211 and D2735). However, the interpretations of this variation are diverse, ranging from a high degree of variability within the genus *Homo* that could include pronounced sexual dimorphism [11], [87], pathological nature [17], [18] or the possible existence of two paleodemes at the site [10], [16], [62]. As we will tackle below, our study has provided some data that would support the latter, as the differences pointed out between D2600 and the other two mandibles are significant, established early in the ontogeny and hardly a consequence of sexual dimorphism or differences in size. The fact that all the specimens were presumably accumulated over a short period of time has often led to discard almost *a priori* the possibility of two populations represented in the Dmanisi sample [9], [11]. However, a detailed analysis of the stratigraphic sequence and the datation of the levels invite to be cautious on this statement, since the time of deposition of the hominin remains could span over an undetermined period of time (see discussion above and Figure 2). In addition, the Dmanisi lithic assemblage also points to the existence of possibly two different technologies and techniques [88], thus supporting the possibility that two paleodemes occupied the same area.

The exceptional difference of height seen between the Dmanisi mandibles (D2600, D2735 and D211) has been interpreted to be an age-related process, given the old age of the individual the mandible D2600 belonged to [17], [18], [89]. It is known that the mandible may experience some growth during adulthood [90]. This (if any) would be small, never reaching the conspicuous height differences ascertained between D2600 and the smaller D21 and D2735. Furthermore, the architectural differences between D211 and D2735, and D2600 we present in our manuscript are not dependant on the length and height of the mandibles.

As noted by Van Arsdale and Lordkipanidze [89] and Martín-Francés et al. [62] the differences in the dental wear gradient along the D2600 molar row has not been observed in any other Pleistocene specimen. According to Margvelashvili and colleagues [18], this severe dental wear in D2600 would have provoked both compensatory eruption of the teeth and remodeling of the alveolar crest, contributing to an increase of the symphysis height. Continuous eruption is one of the three main mechanisms that exist to compensate for enamel wear together with mesial drift and lingual tipping. Compensatory eruption provokes the extrusion of teeth through axial movement, hyperproduction of root cementum and/or remodeling of the socket fundus, but not by increasing the alveolar crest height [63], [91]–[94]. It has been observed in great apes [95], modern humans [63], [96] and earlier hominins [97]. In absence of disease and if the alveolar border is preserved, continuous eruption can be calculated as the distance between the alveolar crest (AC) and the cement enamel junction (CEJ). Compensatory eruption could be also measured as the increasing distance from the mandibular canal to the root apices as suggested by Margvelashvili et al. [18]. However, for doing so, it would be necessary to know the original distance between the mandibular canal and the root apex in absence of compensatory eruption, i.e., to make a longitudinal study within a given group instead of a transversal study across different taxa, since we cannot discern if differences in that distance are attributable to compensatory eruption, to taxonomy or to diet among other factors. In a recent article, Martín-Francés and colleagues [62] observed continuous eruption in the D2600 mandible. The authors calculated a distance between AC and CEJ from 5 to 10 mm depending on the location. As illustrated in Figure 1 of Margvelashvili et al. [18], if the alveolar height in D2600 mandible increased approximately 12 mm, it would mean that teeth needed to erupt from 17 and 22 mm to still keep the 5 to 10 mm distance between AC and CEJ despite the AC growth. Not only these values look excessive but a strong and extensive remodeling of the alveolar socket should be demonstrated with mCT or X-ray images to support the statement. In addition, alveolar changes (if any) as an adaptation to a changing occlusion would provoke indeed the opposite: a recession and thickening of the buccal margin of the alveolar crest known as compensatory receding bone [64], [65]. Thus, we believe that the lack of signs related to periodontal disease, the recorded compensatory eruption and the lack of molar mesial drift are in favour of a stable or minimal alveolar height changes [62].

Skinner et al. [16] investigated the possibility that the high variation observed in the height and breadth of the corpus in D211 and D2600 could be due to sexual dimorphism. According to these authors, the pattern of variation obtained in the Dmanisi mandibles exceeded that of observed in *Gorilla, Pan, Pongo*, and *H. sapiens*. Rightmire et al. [17] criticized this work, and stated that the damage presented by D211 and D2600 in the basal part of the corpus was affecting the measurements taken by Skinner and his team [16]. Furthermore, and according to Rightmire et al. [17] the pathological features exhibited by D2600 may have altered the morphology of this specimen [62]. Besides, other colleagues have also noted the remarkable differences between D2600 and the other mandibles and the crania [70], [98], [99]. Gabounia et al. [19] also suggested that the remarkable differences in the sample could be due to a particular sexual dimorphism in the Dmanisi paleodeme. In this frame, we could explore whether architectural differences between D2600 and the small mandibles presented in our work can be also consequence of sexual dimorphism. In this respect, it has been shown that the spatial relationship between the ramus and the corpus can provide important taxonomical and phylogenetic information [14]. Interestingly, we have noted that the angle measuring this corpus/ramus relationship has a remarkable low variation coefficient in the Atapuerca-SH hominins. The mandibular sample of SH includes both males and females, and very probably all of them belong to the same biological population [100]. Obviously, and although we cannot discard a particular variation in the Dmanisi population, it seems that this feature follows a pattern of primitive/derived condition instead of a pattern of sexual dimorphism.

The small Dmanisi mandibles exhibit a primitive morphological pattern shared with other African hominins and in particular with the early representatives of the *Homo* clade. Thus, the possible relationship of the paleodeme formed by these individuals with *H. habilis* and *H. ergaster* (or African *H. erectus*, according to some authors) seems to be convincing [10], [12], [14]. In contrast, the interpretation of the D2600 specimen is more problematic. It also shows primitive features shared with D211 and D2735, as well as with early *Homo* specimens and even with *Australopithecus* and *Paranthropus*. However, D2600 shows a mosaic of features (Table 3), which seems difficult to understand.

Rosas [48], [53] has performed a detailed study of the morphology and the architecture of the mandibular bone along the European lineage, which includes specimens from the Middle Pleistocene (in particular those of Atapuerca-SH) and the classical Neandertals from the late Middle Pleistocene and the early Upper Pleistocene. In the framework of the craniofacial evolutionary development, Rosas [48] hypothesizes that two distinct and independent morphological processes regulate the variability of the mandible in these hominins. The first process is associated with the backward displacement of features of the corpus, namely the mental foramen, lateral prominence, and anterior marginal tubercle, as well as the anterior border of the ramus. According to Rosas [48], these changes are probably secondary to the spatial position of the mandible in the context of the craniofacial complex. The second process would imply changes in the internal aspect of the corpus and the ramus, mainly the inclination of the mylohyoid line and the alignment of the corpus and ramus, a shallow masseteric fossa, and a deep pterygoid fossa. With respect to the first process, in D2600 the mental foramen and anterior marginal tubercle are placed in a primitive position, whereas the lateral prominence and the anterior border of the ramus are displaced backwards, and there is a conspicuous and subhorizontal retromolar area (despite the great size of the molars and the M1<M2<M3 pattern). This pattern, which combines primitive and derived traits apparently, seems to be incongruous with a process similar to that of proposed by Rosas [48]. With respect to the second process, D2600 shares all the above-mentioned derived features with Neanderthals. In contrast, this mosaic pattern is absent in D211 and D2735.

In relation to these observations, it is interesting to note that some of the most remarkable primitive features of the D2600 mandibles are placed in the infra-nerve unit of the mandible [28] namely, the forward position of the mental foramen, which is placed at the inferior aspect of the corpus [74], the place of the anterior basal tubercle, or the strong projection of the inferior transverse torus. According to Enlow and Hans [24], the structures placed above the alveolar nerve canal differ from those placed under this canal with respect to bone remodeling. Besides that, and according to Rosas and Bastir [28], the alveolar nerve canal contains reflections of the growth process and the properties of mandibular growth rotations. This fact may explain the apparent incongruity of the combination of primitive and derived features observed at the external part of the corpus. The forward position of the lateral protuberance, the alignment of the axis of the corpus and ramus, and the relatively narrow sulcus extramolaris can be related to the posterior relocation of the ramus and corpus and, like in Neanderthals, may be the result of the forward displacement-posterior growth of the mandible [48]. The presence of the large retromolar area in D2600 can be related to a prolonged growth (bone deposition) of the mandibular tuberosity and the resorption of the medial aspect of the ramus. Rosas and Bastir [28] also concluded that the size of the retromolar area represents an allometric variation related to size, and particularly with the supra-nerve unit in samples of chimpanzees, gorillas, modern humans, and (particularly well-expressed) in the Atapuerca-SH hominins and the Neanderthals.

Thus, these features reflect differences strongly linked with patterns of growth and development and can be hardly explained by size or sexual dimorphism. Interestingly, there is another factor we could consider in this analysis. Some authors have suggested that variation in mandibular shape in primates is strongly linked to the mechanical demands of different diets [101]–[106]. If the differences between the Dmanisi mandibles are not the consequence of a particular specific architecture (and the associated specific pattern of growth) then we could hypothesize that these differences are related to particular diets [62]. According to Martín-Francés et al. [62], the wear pattern of D2600 is related to a diet with a high intake of fibrous and abrasive foods such as fruits and plants, similar to the wear pattern recorded in chimpanzees and gorillas. The dentition of D2600 reveals pre- and/or para-masticatory activities such as gripping and stripping and its pattern of wear is different from that recorded in their *Homo* sample. Following this research, there are grounds to explain the presence of two different paleodemes or species in the Dmanisi assemblage, since D2600 could be adapted to a different ecological niche than the rest of the Dmanisi individuals.

In addition, among the dental differences described above between the small Dmanisi mandibles and D2600 such as the size relationship between M1 and M2 is a particularly interesting trait. The M1>M2 sequence is an exception in the whole Early Pleistocene, and the fact that both unique cases come from the same site could ratify that D211 and D2735 belong to the same population, different to that of D2600 where the dental size increases distally. Moreover, we find unlikely that sexual dimorphism works among hominins producing such a molarized bifurcation of the premolar root. While the variability in the premolar’s root number within a human population is usually related to the Tome’s root type, no other examples are found in the hominin fossil record where males develop this markedly bifurcation when compared to females.

In sum, we consider that the small mandibles probably belonged to a paleodeme related to the early African species, *H. habilis* and *H. ergaster*. In contrast, the D2600 mandible could be related to these species but it could also belong to a different and specialized paleodeme. The D2600 mandible exhibits an unexpected and particular mosaic of mandibular features (Table 3) that, in our view, cannot be attributed to the variability (e.g. sexual dimorphism) of a paleodeme that includes D211 and D2735. The relative independence among components of morphological structures (such as the mandible) is a precondition of a mosaic evolutionary change [29]. It is interesting to note in D2600 the retention of primitive traits of the hominin clade together with a set of features that will appear 1.3 million years later in the European hominin lineage. This seems a clear evidence that a similar change in the development pattern could have occurred in hominins remarkably separated in space and time, leading to the appearance of the same set of traits, probably as a case of homoplasy. We can assume that the same development process has not operated in the Neanderthals and in D2600, given the strong craniofacial differences between the skull of Neanderthals and the skull 5 from Dmanisi. The morphology of D2600 responds to a specific growth process, which led to combination of features never found in other hominins. The derived traits have to be combined with primitive features in a harmonious mosaic pattern in order to perform the corresponding function [35]. It is interesting that some measurements and indices obtained in D2600 are out of the variability of *H. habilis*, *H. ergaster*, and *H. erectus*, contra conclusions of [11], adding more uncertainty to the phylogenetic relationships of this enigmatic specimen.

With regard to the present observations, we believe that the definition of a new paleodeme (species) [15] is justified. If we consider that the differences between the skull 5 from Dmanisi and the other specimens are enough to consider the species level, then the nomen *H. georgicus* would represent a valid taxon. Our results suggest that the individuals represented by D211 and D2735 might belong to a different paleodeme (species), with a completely distinct craniofacial growth pattern. Furthermore, according to Baena and colleagues [88] the lithic assemblage recovered from the Dmanisi site could be also congruent with the existence of two different populations.

We expect that future discoveries at Dmanisi and revisions of the Plio-Pleistocene hominin fossil record can help to shed light in the taxonomic and phylogenetic interpretation of these hominins. However, the evidence available at present suggests that the scenario of the first dispersion out of Africa was probably more complex than what it was previously supposed. Our own analysis on the original specimens suggests that different ecological niches may have been present in the area where the Dmanisi fossils were found. The possibility of two paleodemes represented in the Dmanisi hypodigm should be further explored.
